# Fortetropin inhibits disuse muscle atrophy in dogs after tibial plateau leveling osteotomy

**DOI:** 10.1371/journal.pone.0231306

**Published:** 2020-04-09

**Authors:** Dana A. White, Kenneth R. Harkin, James K. Roush, Walter C. Renberg, David Biller

**Affiliations:** Department of Clinical Sciences, Veterinary Health Center, Kansas State University, Manhattan, Kansas, United States of America; University of Tennessee Health Science Center College of Graduate Health Sciences, UNITED STATES

## Abstract

**Objective:**

To determine if a commercial myostatin reducer (Fortetropin®) would inhibit disuse muscle atrophy in dogs after a tibial plateau leveling osteotomy.

**Design:**

A prospective randomized, double-blinded, placebo-controlled clinical trial.

**Animals:**

One hundred client-owned dogs presenting for surgical correction of cranial cruciate ligament rupture by tibial plateau leveling osteotomy.

**Procedures:**

Patients were randomly assigned into the Fortetropin® or placebo group and clients were instructed to add the assigned supplement to the dog’s normal diet once daily for twelve weeks. Enrolled patients had ultrasound measurements of muscle thickness, tape measure measurements of thigh circumference, serum myostatin level assays, and static stance analysis evaluated at weeks 0, 8, and 12.

**Results:**

From week 0 to week 8, there was no change for thigh circumference in the Fortetropin® group for the affected limb (-0.54cm, P = 0.31), but a significant decrease in thigh circumference for the placebo group (-1.21cm, P = 0.03). There was no significant change in serum myostatin levels of dogs in the Fortetropin® group at any time point (P>0.05), while there was a significant rise of serum myostatin levels of dogs in placebo group during the period of forced exercise restriction (week 0 to week 8; +2,892 pg/ml, P = 0.02). The percent of body weight supported by the affected limb increased in dogs treated with Fortetropin® (+7.0%, P<0.01) and the placebo group (+4.9%, P<0.01) at the end of the period of forced exercise restriction. The difference in weight bearing between the Fortetropin® and placebo groups was not statistically significant (P = 0.10).

**Conclusion:**

Dogs receiving Fortetropin® had a similar increase in stance force on the affected limb, no significant increase in serum myostatin levels, and no significant reduction in thigh circumference at the end of the period of forced exercise restriction compared to the placebo. These findings support the feeding of Fortetropin® to prevent disuse muscle atrophy in canine patients undergoing a tibial plateau leveling osteotomy.

## Introduction

Loss of lean body mass can result from cachexia, sarcopenia, and disuse atrophy. Cachexia is the loss of lean body mass as a result of catabolism secondary to a disease state [1]. Sarcopenia, an age-related reduction in muscle mass, occurs in the absence of disease [2]. The loss of lean body mass due to disuse muscle atrophy can result from immobilization or inactivity associated with recovery from surgery or reduction in the use of a limb secondary to pain [3]. While the trigger for muscle loss varies between these three processes, increased expression of myostatin appears to be a common link [1–3].

During periods of immobilization, disuse atrophy occurs because increased myostatin mRNA expression results in both an increase in muscle protein breakdown and a reduction in muscle protein synthesis [4]. The effect on muscle atrophy can be global, as myostatin acts via autocrine, paracrine, and endocrine pathways [4]. The resultant increase in serum myostatin levels associated with the endocrine pathway can be measured and reduction of these levels is a potential target for prevention of disuse atrophy. The use of a canine-specific activin receptor type IIB decoy receptor in dogs did not reverse cardiac cachexia, but reduction of serum myostatin by an insertion of a canine myostatin propeptide gene utilizing an adeno-associated virus serotype 8 vector increased muscle mass in both healthy dogs and golden retrievers with muscular dystrophy [5–7].

Fortetropin®, a non-thermal pasteurized, freeze-dried, fertilized, egg yolk product, is considered a natural myostatin-reducing agent. When administered to healthy adults, Fortetropin® reduced serum myostatin levels by 18–22% and increased lean mass and muscle thickness in comparison to a placebo [8]. In that same study, Fortetropin® administered to rats prevented the rise in ubiquination of proteins and increased mTOR signaling, consistent with a reduction in serum myostatin [8].

Cranial cruciate ligament (CCL) rupture is the most common stifle injury in medium and large breed dogs, ultimately resulting in pain, lameness, and secondary muscle atrophy of the affected limb [9]. While various surgical procedures are described for the repair of CCL rupture, the tibial plateau leveling osteotomy (TPLO) is the most common repair technique for the cranial cruciate ligament deficient stifle, with the ultimate goal of providing functional stifle joint stability [10–13]. There have not been any studies addressing the effect of disuse muscle atrophy on stifle joint stability or on interventions to mitigate disuse muscle atrophy in dogs with CCL rupture.

The purpose of this study was to assess if a myostatin reducer would have an effect on preventing or reversing disuse muscle atrophy in patients undergoing a tibial plateau leveling osteotomy. Our hypothesis was that Fortetropin® supplementation in the post-operative period would help reverse disuse muscle atrophy in the affected limb, resulting in improved force distribution at standing and measurable difference in muscle thickness at the rechecks compared to dogs receiving the placebo.

## Materials and methods

### Animals

One hundred client-owned dogs with naturally-occurring cranial cruciate ligament rupture were randomized into one of two groups: a Fortetropin® treatment group or a placebo-control group. All assessments as described were performed on each dog preoperatively, and at 8 and 12 weeks postoperatively.

### Inclusion criteria

Between May 2017 and September 2018, a TPLO was performed on 254 dogs with naturally-occurring CCL rupture at the Veterinary Health Center at Kansas State University. For inclusion in the study, dogs had to weigh at least 18kg and no more than 75kg, have a diagnosis of naturally- occurring cranial cruciate ligament rupture, be apparently healthy prior to the ligamentous rupture, and have no concurrent disease identified on physical examination or on pre-operative bloodwork. Each patient underwent general anesthesia for completion of a routine tibial plateau leveling osteotomy (TPLO) [9]. Patients with bilateral cranial cruciate ligament rupture were included in the study; however, after a TPLO was performed, surgical correction of the second (contralateral) limb was not performed until completion of the 12-week study. Exclusion criteria included orthopedic or neurologic comorbidities that might affect weight bearing, surgical correction of the contralateral cranial cruciate ligament within 6 months prior to enrollment, revision of a previous ipsilateral repair for cranial cruciate ligament rupture, and owner reported allergies to egg products. Patients were enrolled if they met the inclusion criteria and owners consented to two follow up appointments at 8 weeks and 12 weeks post-operatively.

The study was approved by the Kansas State University Institute Animal Care and Use Committee. Owners of the dogs agreed to have their animals participate in the study and signed a statement of informed consent.

### Supplementation

The Fortetropin® (MYOS Rens Technology (Cedar Knolls, NJ)) and placebo supplements were provided by Myos Rens Technologies, and randomly assigned to each dog with use of a published random numbers table [14]. Clients were provided the powdered supplement in a light-resistant package labeled “A” or “B” at the time of discharge. The dogs were administered Fortetropin®, a freeze-dried, nonthermal pasteurized, chicken egg yolk product (product A) or the placebo which was a cheese powder (product B), with the test agents designed to closely matched macronutrient composition. Both clients and investigators were blinded to the treatment and placebo agent. The Fortetropin® composition data was 55% fat, 33% protein, and 7% carbohydrate, while the cheese powder (placebo) was 48% fat, 36% protein, and 4% carbohydrate. Dogs were dosed at 300mg/kg daily (one 6600mg scoop/22kg), and the assigned supplement was dosed to the closest ½ scoop without under dosing. The owners were instructed to feed the provided supplement once daily for 12 weeks and the powdered supplement could be mixed with canned food to facilitate ingestion.

### Patient evaluation

The following data were obtained at baseline (week 0), at the end of the period of forced exercise restriction (week 8), and at the end of the period of gradual return to full exercise (week 12): thigh circumference, ultrasonographic measurements of thigh muscle thickness, stance force analysis, and serum myostatin levels.

Thigh circumference was measured by one of two investigators (DW or JKR) with a single retractable tape measure device at the mid-point of the femur for both the affected (leg on which the TPLO was performed) and unaffected (contralateral) leg. The measurement was recorded as affected thigh circumference (cm), ATC, or unaffected thigh circumference (cm), UATC.

B-mode ultrasonography (Toshiba Aplio, 500, Toshiba Medical Systems, Japan) was used to determine the muscle thickness cranial to the mid-femur and lateral to the mid femur of both the affected and contralateral hindlimbs. The patients were placed in lateral recumbency with the stifle and tarsus held at 90°. The hair over the mid-point of the femur was clipped prior to obtaining the measurements of the lateral and cranial muscles of the uppermost leg. Measurements (mm) were obtained and recorded by a board-certified radiologist, radiology resident, or radiology intern as affected cranial muscle thickness (ACr), affected lateral muscle thickness (ALat), unaffected cranial muscle thickness (UACr), and unaffected lateral muscle thickness (UALat) (Table 1). B-mode ultrasonography was also used to measure the dorsal to ventral transverse thickness of the epaxial muscles at the level of the 13^th^ rib with the dogs in sternal recumbency, after clipping the hair, for both the affected and unaffected sides as previously described [2]. Measurements (mm) were recorded as affected epaxial muscle (AEpM) and unaffected epaxial muscle (UAEpM) (Table 1).

**Table 1:** Abbreviations used for muscle measurements.

Abbreviation	Term	Measurement
ACr	Affected cranial thickness	mm
ALat	Affected lateral thickness	mm
ATC	Affected thigh circumference	cm
AEpM	Affected epaxial muscle	mm
UACr	Unaffected cranial thickness	mm
UALat	Unaffected lateral thickness	mm
UATC	Unaffected thigh circumference	cm
UAEpM	Unaffected epaxial muscle	mm

For stance analysis, the percent body weight placed on each limb at a static stance was recorded with a PetSafe Stance Analyzer (Stance Analyzer, LiteCure Companion Animal Health (Newark, DE)) for all dogs prior to sedation. Measurements (% body weight) were recorded as ipsilateral front limb (IPSFL), contralateral front limb (COFL), affected hindlimb (AHL), and contralateral hindlimb (COHL).

For serum myostatin measurements, 3-6ml of blood was collected from a peripheral vein and placed in a BD Vacutainer® serum blood collection tube. The serum was routinely separated after clot formation. Serum was stored at -80° C until the time of the assay. Batched samples were thawed and serum myostatin concentrations were measured in duplicate using a GDF-8 Myostatin Quantikine ELISA kit (Quantikine ELISA, GDF-8/Myostatin Immunoasay, R&D Systems (Minneapolis, MN)) according to the manufacturer’s instructions, with the following exception: after running the first 96-well plate of samples at the manufacture’s recommended 1:20 dilution, a number of values were outside the control range, so a 1:25 dilution was performed on the second 96-well plate, and then for all remaining samples a 1:30 dilution was performed. All samples that initially were outside the control range were repeated at 1:30 dilutions.

### Statistics

Statistical analysis was performed by an independent statistical firm (Dr. Murari Singh, DACS PLUS (Ontario, Canada)). Variables measured were defined as: stress index (SI = (difference of measurement in week 8 from that in week 0)/8), recovery index (RI = (difference of measurement in week 12 from that in week 8)/4), and overall atrophy index (AI = (difference of measurement in week 12 from that in week 0)/12).

Statistical assessment of these indices was carried out for each time period using independent group t-test for comparison within each product and analysis of variance (ANOVA) for comparison between the two supplements (Fortetropin® and placebo). Residual plots were examined to check for any serious departure from the ANOVA assumptions. For measures during the SI or RI with expected decreases over time (e.g., thigh circumference, muscle thickness during the SI period) or increases (e.g., myostatin during the SI period), testing significance was performed using, one-sided tests. Where the direction of change was not implied by the biological factors (e.g., for AI and myostatin levels), two-sided tests were used. P-values were reported for the probability of obtaining more extreme data than observed under the null hypothesis of zero mean or no difference between means for Fortetropin® and the placebo. The level of statistical significance was set at 5% (p≤ 0.05). Results with a p value between 0.05 and 0.1 were reported in the event of a type II error. GenStat software (GenStat software, VSN International (Hempstead, UK)) was used to carry out the analyses.

## Results

### Dogs

One hundred dogs met the inclusion criteria. The most common breeds represented were Labrador retriever (n = 23), Labrador retriever mix (n = 9), golden retriever (n = 9), boxer (n = 5), American bulldog (n = 4), rottweiler (n = 4), American Staffordshire terrier (n = 3), American Staffordshire terrier mix (n = 3). The remaining 40 dogs were distributed over 31 different breeds of mixed breeds. There were 71 female dogs (66 spayed) and 29 male dogs (27 castrated). Forty-two dogs presented with a rupture of the right CCL, 42 dogs presented with a rupture of the left CCL, and 16 dogs presented with bilateral CCL ruptures. Of the dogs with bilateral CCL ruptures (9 dogs in the Fortetropin® group and 7 in the placebo group) the TPLO was performed on the right in 9 dogs and on the left in 7 dogs. Two dogs had undergone a TPLO on the contralateral limb at least 6 months prior to enrollment. Two dogs ruptured the contralateral CCL prior to the 8-week recheck and underwent a TPLO after completion of the study (1 dog in the Fortetropin® group and 1 dog in the placebo group). One dog in the Fortetropin® group ruptured the contralateral CCL at 9 weeks post-operatively, and underwent surgery at the 12-week recheck. Five dogs with contralateral CCL rupture confirmed at the time of enrollment had a contralateral TPLO performed after completion of the study.

Fourteen dogs did not return for evaluation at the 8-week time period; although, one of these dogs did return for the 12-week recheck. Reasons for dropping from the study included: lost to follow up (n = 7), development of diarrhea (n = 3), development of surgical site infection one week post-operatively (n = 2), anxiety at veterinary visits (n = 1), and death after being hit by a car (n = 1). Nine additional dogs did not complete the 12-week follow up. Reasons for dropping the last visit included: lost to follow up (n = 6), meniscal tear (n = 1), owner discontinuation of supplement early (n = 1), and conjunctivitis and pruritus (n = 1).

Complete data sets for stance analysis and muscle thickness measurements were obtained for 77 dogs and 69 dogs had complete data sets for serum myostatin. From week 0 to week 8, 85 data sets were available for stance analysis and muscle thickness, while 81 data sets were available for myostatin (as four dogs had week 0 and week 12 myostatin data but no week 8). There were 14 dogs with no stance analysis or muscle thickness measurements at weeks 8 or 12 (9 in the Fortetropin® group and 5 in the placebo group). There were 15 dogs with no myostatin measurements (9 in the Fortetropin® group and 6 in the placebo group).

Mean ± SD age of all dogs enrolled at the time of surgery was 5.48 ± 2.48 years (range, 1.25 to 12.25 years), and mean body weight was 34.25 ± 8.69kg (range, 19 to 71kg). The TPLOs were performed by one of seven different doctors including two ACVS diplomats, one small animal surgical residency trained clinician, and four small animal surgery residents. Fifty-two dogs were randomly allocated to receive Fortetropin® and 48 dogs received the placebo.

### Within group: Fortetropin®

Dogs receiving Fortetropin® had no significant change in thigh circumference (ATC or UATC) over any of the time periods (Table 2). There was a significant decrease for ACr (mean 4.49mm), UACr (3.44mm), ALat (1.44mm), AEpM (2.15mm), UAEpM (2.18) from week 0 to week 8, but not from weeks 0–12 or weeks 8–12. Stance analysis showed a significant increase in percent weight supported by the AHL from 0–8 weeks (mean 5.74%) and 0–12 weeks (mean 7.00%), but not 8–12 weeks (mean 1.26%). There was a significant reduction in percent weight supported by the COHL from 0–8 weeks (4.3%) and 0–12 weeks (6.5%), but not 8–12 weeks (2.2%) (Table 3). There was no significant change in serum myostatin levels over any time period (45.1pg/ml/week, -207.7pg/ml/week, and -43pg/ml/week, respectively for weeks 0–12, 8–12, and 0–12).

**Table 2:** P-values, of changes over week 0 to week 12 in the atrophy and biochemical parameters in dogs under two nutrient formulations.

		Week8—Week0			Week 12- Week 8			Week 12- Week0		
		Within Fortetropin®	Within Placebo	Between Fortetropin® and Placebo	Within Fortetropin®	Within Placebo	Between Fortetropin® and Placebo	Within Fortetro-pin®	Within Placebo	Between Fortetrop-in® and Placebo
a) Muscle atrophy	ATC (cm)	0.31	0.03	0.29	0.50^$^	0.18	0.36	0.47	0.89	0.66
	UATC (cm)	0.37	0.02	0.09	0.50^$^	0.18	0.26	0.38	0.15	0.68
	ACr (mm)	0.00	0.05	0.39	0.35	0.50^$^	0.34	0.04	0.03	0.83
	UACr (mm)	0.01	0.11	0.27	0.15	0.46	0.36	0.19	0.12	0.96
	ALat (mm)	0.04	0.12	0.66	0.13	0.04	0.45	0.81	0.86	0.93
	UALat (mm)	0.08	0.21	0.64	0.30	0.28	0.89	0.87	0.47	0.67
	AEpM (mm)	0.01	0.36	0.12	0.20	0.06	0.60	0.30	0.35	0.11
	UAEpM (mm)	0.00	0.46	0.06	0.12	0.50^$^	0.36	0.18	0.91	0.34
b) Blood Marker	Myostatin (pg/ml)	0.33	0.02	0.08	0.28	0.26	0.94	0.7	0.15	0.12
c) Stance Analysis	IPSFL (%)	0.12	0.99	0.28	0.25	0.67	0.59	0.93	0.58	0.71
	COFL (%)	0.44	0.90	0.53	0.59	0.75	0.52	0.68	0.79	0.60
	AHL (%)	<0.01	0.01	0.10	0.37	0.10	0.56	<0.01	<0.01	0.31
	COHL (%)	<0.01	0.03	0.36	0.12	<0.01	0.38	<0.01	<0.01	0.97
	WT (kg)	0.84	0.83	0.95	0.92	0.33	0.50	0.51	0.25	0.61

^$^: Bound where the P-value exceeded 0.5 in case of one-tail tests (due to measurement errors and or inappropriate alternative hypothesis).

**Table 3:** Mean values of atrophy and biochemical parameters under the two supplements.

		Fortetropin®			Placebo		
		Week0	Week8	Week12	Week0	Week8	Week12
a) Muscle atrophy	ATC (cm)	34.24	33.70	33.28	33.74^a^	32.53	33.48
	SE	0.62	0.63	0.73	0.63	0.64	0.68
	UATC (cm)	36.51	35.80	35.16	36.30^a^	34.26	35.35
	SE	0.70	0.85	0.78	0.72	0.88	0.73
	ACr (mm)	25.62	21.13^a^	21.12^a^	25.34^a^	22.78	22.17^a^
	SE	1.21	1.25	1.10	1.26	1.29	1.03
	UACr (mm)	27.70	24.26^a^	24.56	27.26	25.38	25.60
	SE	1.17	1.16	1.24	1.21	1.20	1.19
	ALat (mm)	16.39	14.95^a^	15.41	16.17	15.24	16.23^b^
	SE	0.64	0.71	0.68	0.67	0.73	0.63
	UALat (mm)	21.15	19.61^a^	19.70	21.02	20.26	20.29
	SE	0.78	0.69	0.89	0.81	0.71	0.83
	AEpM (mm)	23.65	21.50^a^	21.91	21.82	21.36	22.37^b^
	SE	0.65	0.66	0.69	0.67	0.69	0.65
	UAEpM (mm)	22.94	20.76^a^	21.40	22.05	21.82	21.78
	SE	0.61	0.57	0.70	0.63	0.59	0.66
b) Blood Marker	Myostatin (pg/ml)	29861	30684	30041	29334^a^	32226	32133
	SE	1754	1974	2007	1775	1998	1874
c) Stance Analysis	IPSFL (%)	33.00	30.76^a^	32.50	32.96	32.84	33.44
	SE	0.98	0.90	1.04	1.02	0.93	1.01
	COFL (%)	31.31	32.30^a^	31.50	31.60	31.21	31.98
	SE	1.10	1.03	1.22	1.15	1.07	1.17
	AHL (%)	8.00	13.74	15.00^a^	9.63^a^	12.88	14.54^a^
	SE	0.87	0.83	0.96	0.90	0.86	0.93
	COHL (%)	27.50	23.20	21.00^a^	25.81^a^	23.07	20.05^a^^,^^b^
	SE	1.11	1.07	1.15	1.15	1.11	1.11
	WT (kg)	78.24	77.52	76.44	73.76	72.92	74.49
	SE	2.96	3.24	3.62	3.08	3.39	3.49

SE = estimated standard error of the mean in the preceding row

^a^ significantly different from week 0 at p<0.05

^b^ significantly different from week 8 at p<0.05

### Within group: Placebo

Dogs receiving the placebo, had a significant reduction in thigh circumference (ATC and UATC) (Table 2) from week 0–8 (mean 1.21cm and 2.04cm, respectively (Table 3)). There was no significant change in UACr, ALat, UALat, AEpM and UAEpM over any time period (Table 2). Stance analysis showed a significant increase in percent weight supported by the AHL (mean 2.25% and 4.91%, respectively for weeks 0–8 and 0–12) and a significant decrease in percent weight supported by the COHL over all time periods (2.74%, 3.02%, and 5.76%, respectively for weeks 0–8, 8–12, and 0–12) (Tables 2 and 3). There was a significant increase in serum myostatin levels from week 0–8 (358.0pg/ml/week) but not from week 8–12 (-244.7pg/ml/week) or from week 0–12 (185.9pg/ml/week).

### Fortetropin® compared to placebo

There were no differences between the means of the treatments for decrease, increase, or change (SI, RI, and AI based on the pairs of measurement weeks) for all remaining parameters.

## Discussion

The TPLO is the most common orthopedic surgery for stabilization of cranial cruciate ligament deficient stifles in dogs [12,13]. Patients undergoing a TPLO and other orthopedic procedures are often subjected to activity restriction, and the development of disuse muscle atrophy following orthopedic injuries has been documented [15]. Our study looked at the use of the Fortetropin® supplementation to reverse or inhibit disuse muscle atrophy in dogs affected with cranial cruciate ligament disease and receiving a TPLO. The addition of Fortetropin® to diets following TPLO resulted in a positive change in thigh circumference compared to dogs receiving the placebo. Both groups had increases in percent weight bearing during the period of forced activity restriction, with the difference between the two groups nearing significance (P = 0.10).

Percent weight bearing with use of stance analysis has been evaluated for dogs undergoing TPLO [16,17]. A recent publication reported no effect on weight bearing when evaluating surgeon, surgical experience, arthrotomy, meniscal damage, meniscal intervention, complications, postoperative TPA, and initial TPA in dogs undergoing TPLO [16]. Our study showed similar increases in percent of weight supported in the affected hind limb in dogs treated with Fortetropin® than the placebo in the period of forced exercise restriction (week 0 to week 8), with the Fortetropin® group nearly reaching significance over the placebo group (P = 0.10). One reason dogs receiving Fortetropin® may not have had a significant increase in percent weight bearing in the week 8 to week 12 period is that by week 8 they were equivalently where placebo dogs were by week 12; therefore, there was not an expectation for an increase in week 8 to week 12 in Fortetropin® dogs.

Serum myostatin levels in the dogs in our study were not significantly changed in the Fortetropin® group but were significantly increased in the placebo supplemented dogs during the period of forced exercise restriction. Similarly, in a study evaluating the effects of neuromuscular electrical stimulation to inhibit disuse muscle atrophy in immobilized limbs in humans, serum myostatin levels rose significantly in the untreated group but did not change in the treated group [18]. Preventing the rise in serum myostatin during periods of immobilization or exercise restriction appears to be a critical factor in minimizing disuse muscle atrophy.

Although there was a significant decrease in thigh circumference for the placebo group for the affected limb from week 0–8 and the unaffected limb from week 0–8, there was no significant change in thigh circumference in the Fortetropin® group. The finding supports the conclusion that Fortetropin® inhibited disuse muscle atrophy during the period of forced exercise restriction and is consistent with the results of the myostatin measurements. In contrast to the thigh circumference measurement, the majority of ultrasound measurements of muscle thickness were significantly decreased from week 0–8 in the Fortetropin® group and decreased but not significantly in the placebo group. Although a reduction in thigh muscle mass is expected following transection of the cranial cruciate ligament for up to 5 weeks following immediate stabilization of the joint, these results were not consistent with the measurement of thigh circumference for the dogs in the current study [18].

Although a recent study concluded that ultrasonographic measurement of muscle thickness compared favorable to computed tomographic measurements in dogs [19], we identified several possible limitations in our study related to the use of ultrasonography to measure muscle thickness. We were unable to ensure that the same radiology personnel performed all ultrasonographic measurements, and inter-observer variation was evident even on the same dogs at the same visit and was considered a limitation. This variation was attributed to various degrees of force applied by the different examiners on the muscle mass being evaluated. In the absence of a force transducer, this could not be standardized for each measurement and excessive pressure placed on the muscle through the probe likely resulted in tissue distortion and an underestimation of depth during image acquisition [20].

It is also possible that thigh circumference measurement was unreliable. In this study we did not randomize for breed-matched cases or hair coat thickness and hair thickness has previously been reported to affect thigh circumference measurements in dogs [21]. Since baseline measurements were taken prior to aseptic preparation of the affected limb for completion of the TPLO, variable rates of hair regrowth following surgery could result in perceived changes when using a spring tension measuring device [20]. However, given that thigh circumference measurements were taken by one of two individuals, we felt these measurements were more consistent and reliable throughout the study than ultrasonographic measurements of muscle thickness.

Another limitation was the lack of activity monitoring of patients during the restricted exercise period, as activity restriction was based on owner compliance.

Fortetropin® has previously been shown to increase lean body mass and decrease myostatin levels in humans [8]. This study is the first published report of the use of Fortetropin® in dogs undergoing orthopedic surgery. During the period of forced exercise restriction from week 0 to week 8, when comparing Fortetropin® supplementation to a macronutrient placebo, Fortetropin® prevented a rise in serum myostatin levels and prevented the loss of muscle mass, as measured by thigh circumference in the affected and unaffected limbs. Therefore, the use of Fortetropin® could be considered as a beneficial supplement in the perioperative period for patients undergoing elective orthopedic procedures.
